# Prolonged Starvation Causes Up-Regulation of AQP1 in Adipose Tissue Capillaries of AQP7 Knock-Out Mice

**DOI:** 10.3390/ijms17071101

**Published:** 2016-07-22

**Authors:** Mariusz T. Skowronski, Agnieszka Skowronska, Aleksandra Rojek, Michal K. Oklinski, Søren Nielsen

**Affiliations:** 1 Department of Animal Physiology, University of Warmia and Mazury in Olsztyn, Olsztyn 10-752, Poland; 2 Department of Human Physiology, University of Warmia and Mazury in Olsztyn, Olsztyn 10-752, Poland; agnieszka.skowronska@uwm.edu.pl; 3 Department of Health Science and Technology, Aalborg University, Aalborg 9220, Denmark; aleksandra_rojek@yahoo.com (A.R.); mko@hst.aau.dk (M.K.O.); sn@hst.aau.dk (S.N.)

**Keywords:** AQP1, protein expression, fasting, immunolocalization, immunoelectron microscopy, AQP7 knock-out mice

## Abstract

Aquaporins (AQPs) are membrane proteins involved in the regulation of cellular transport and the balance of water and glycerol and cell volume in the white adipose tissue (WAT). In our previous study, we found the co-expression of the AQP1 water channel and AQP7 in the mouse WAT. In our present study, we aimed to find out whether prolonged starvation influences the AQP1 expression of AQP7 knock-out mice (AQP7 KO) in the WAT. To resolve this hypothesis, immunoperoxidase, immunoblot and immunogold microscopy were used. AQP1 expression was found with the use of immunohistochemistry and was confirmed by immunogold microscopy in the vessels of mouse WAT of all studied groups. Semi-quantitative immunoblot and quantitative immunogold microscopy showed a significant increase (by 2.5- to 3-fold) in the abundance of AQP1 protein expression in WAT in the 72 h starved AQP7 KO mice as compared to AQP7+/+ (*p* < 0.05) and AQP7−/− (*p* < 0.01) controls, respectively. In conclusion, the AQP1 water channel located in the vessels of WAT is up-regulated in response to prolonged starvation in the WAT of AQP7 KO mice. The present data suggest that an interaction of different AQP isoforms is required for maintaining proper water homeostasis within the mice WAT.

## 1. Introduction

Aquaporins (AQPs) are membrane proteins engaged in the transport of water and small solutes which play a wide multiplicity of important physiological roles. The AQP family can be classified into the orthodox aquaporins (AQP0, AQP1, AQP2, AQP4, and AQP5), aquaglyceroporins (AQP3, AQP7, AQP9, and AQP10), and the unorthodox aquaporins (AQP6, AQP8, AQP11, and AQP12) [1]. While all AQPs are permeable to water, the aquaglyceroporins are permeable to glycerol and other small solutes such as urea, purines and arsenite [1]. It is typical that multiple AQP isoforms are present in a single cell and the adipose cell is no exception, where several members of this family have been identified. However, the importance of overlapping AQP expression is unclear, although interisoform interactions might be required for proper cellular functions [2]. To date, several mammalian AQP family members have been identified in the white adipose tissue (WAT), including: AQP1, AQP3, AQP5, AQP7, AQP10, and AQP11 [1,2,3,4].

AQP1 is considered to be a water-selective channel and it allows the cell to maintain an appropriate water balance for its proper function in response to osmotic gradients. It has also been reported that AQP1 transports ions and gases (i.e., carbon dioxide ammonia and nitric oxide) [5].

AQP1 is present throughout the microvasculature of endothelial cells, including kidneys, secretory glands, lungs, airways, muscles, peritoneum and pleura [6,7,8]. Moreover, AQP1 was also seen in endothelial cells in the lacteals of the small intestine and cornea [6,9]. Furthermore, Mobasheri and Marples [10] found the presence of AQP1 mRNA in endothelial and epithelial cells including the renal cortex, pancreatic ducts, liver, gallbladder, choroid plexus, and ependymal cells of the central nervous system. Moreover, Saadoun et al. [11] proposed that AQP1 is an important initiator of cell migration and angiogenesis.

AQP7 is mainly expressed in WAT [12] and its function appears to be related to the transport of glycerol through plasma membranes. Our knowledge of the potential role of AQP7 has been emphasized by the use of AQP7 knock-out mice (AQP7 KO). Recently, we and other groups [4,13,14] have generated AQP7 KO mice using a different method and, interestingly, the mice show different phenotypes. The significant difference between the phenotypes of KO mice was in their susceptibility to developing obesity and cellular localization of AQP7 in adipocytes and capillaries of WAT. The expression of AQP7 protein in the capillaries, but not in the surrounding adipocytes of mouse WAT, suggests a non-cell-autonomous role for this protein in promoting antihypertrophic effects [4]. A recent study performed by Miyauchi et al. [15] found that AQP7 is expressed in capillaries at a higher level than in adipocyte plasma membranes in mouse.

Our previous study [4] showed a co-expression of AQP7 with AQP1 in the capillary endothelia of mouse WAT. Therefore, in this study, we aimed to investigate: (1) the cellular localization of AQP1 by immunoperoxidase; (2) the expression of AQP1 protein by semi-quantitative immunoblotting; (3) sub-cellular localization and expression of AQP1 by quantitative immuno-gold electron microscopy in WAT of AQP7 KO mice after prolonged fasting.

## 2. Results

**Figure 1:**
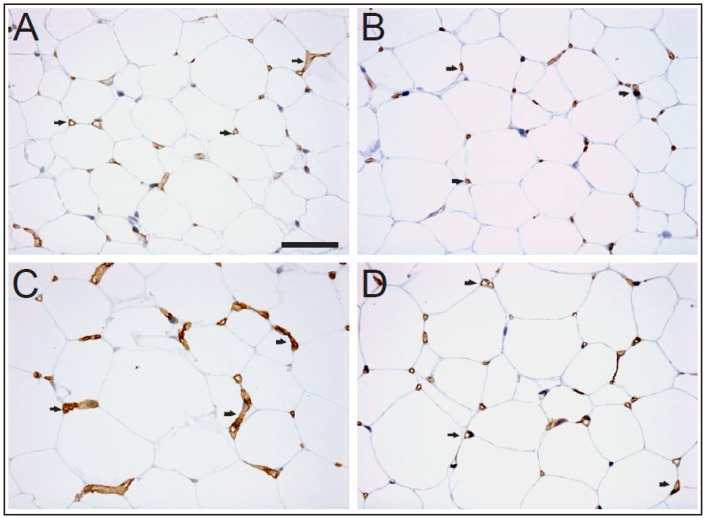
AQP1 expression in capillary endothelia of mouse WAT. Immunohistochemical staining of AQP1 in paraffin-embedded sections of the WAT from control and fasted mice (control: AQP7−/− (**A**); AQP7+/+ (**B**); fasted: AQP7−/− (**C**); AQP7+/+ (**D**)). Anti-AQP1 antibody labels small capillaries of WAT in control (**A**,**B**) and fasted (**C**,**D**) mice. Arrows indicate localization of AQP1 in mouse WAT. A bar indicates 50 µm.

**Figure 2:**
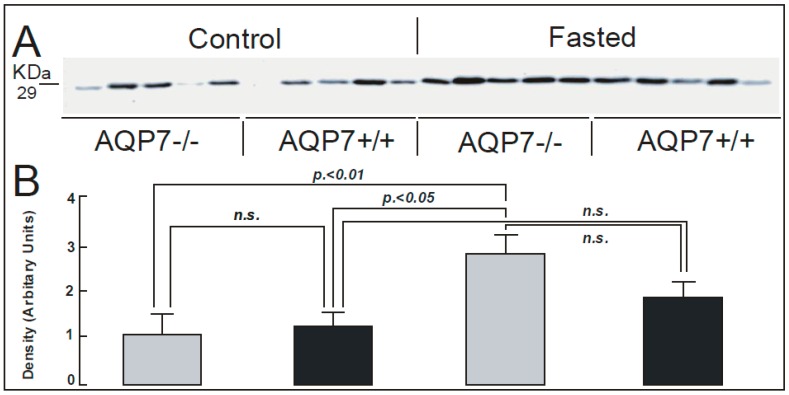
Western blot analysis of AQP1 protein expression in capillary endothelia of WAT from control and fasted mice (control: AQP7−/−, AQP7+/+; fasted: AQP7−/−, AQP7+/+). Anti-AQP1 antibody recognizes a ~29 kDa band in membrane fractions from mouse WAT (**A**). Densitometric analysis of AQP1 was performed and normalized against total protein amount (**B**).

**Figure 3:**
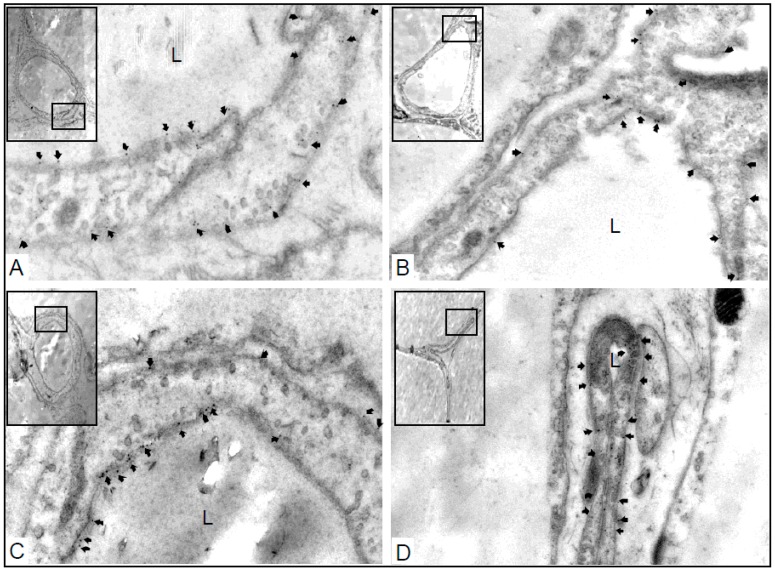
Immunoelectron-microscopic localization of AQP1 in an ultrathin Lowicryl section of peri-epididymal WAT capillaries from control and fasted mice (control: AQP7−/− (**A**); AQP7+/+ (**B**); fasted: AQP7−/− (**C**); AQP7+/+ (**D**)). Both apical and basal plasma membranes exhibit labeling (arrows, **A**–**D**). Magnification; ×28,000; L; lumen.

**Figure 4:**
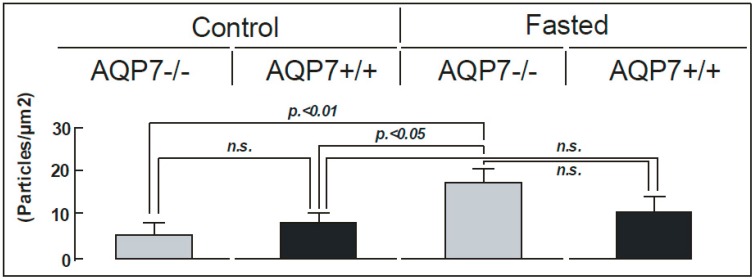
Numbers of gold particles/µm^2^ in capillary endothelia of WAT from control and fasted mice (control: AQP7−/−, AQP7+/+; fasted: AQP7−/−, AQP7+/+). Quantitation of the electron micrograph results was performed using the iTEM, FIVE Digital Imaging Solutions, based on the analySIS Platform (Olympus, Tokyo, Japan), number of areas counted (*n* = 20) for each mean ± SD.

In this study, AQP7 knock-out mice were employed in immunohistological (Figure 1), semi-quantitative immunoblotting (Figure 2) and quantitative immuno-gold electron microscopy (Figure 3 and Figure 4) studies to clarify the profile of AQP1 expression in the WAT vessels. By immunohistological study at the light microscopic level (Figure 1), we found AQP1 to be expressed in WAT vessels of all genotypes of mice used in this study (control: AQP7−/−, AQP7+/+ and fasted: AQP7−/−, AQP7+/+). We found that prolonged (72 h) fasting increased the immunoreactivity of AQP1 in AQP7 KO (Figure 1C) and AQP7+/+ mice (Figure 1D) when compared to the respective controls (Figure 1A,B). Prolonged fasting of AQP7−/− and AQP7+/+ (Figure 1C,D) had no evident effect on adipocyte size when compared to the controls (Figure 1A,B). Moreover, the abundance of AQP1 in vessels was regulated in response to starvation (Figure 2). Western immunoblot with the mouse antibody against AQP1 recognized a 29 kDa band in WAT of the control (AQP7−/−, AQP7+/+) and fasted (AQP7−/−, AQP7+/+) mice (Figure 2A). Semi-quantitative immunoblot (Figure 2B) showed 2.5- to 3-fold increase in the white adipose tissue AQP1 protein level in the 72 h starved AQP7 KO mice as compared to AQP7−/− and AQP7+/+ controls, with *p* < 0.01 (*n* = 5) and *p* < 0.05 (*n* = 5), respectively.

Finally, the sub-cellular localization of AQP1 in capillaries was determined by immuno-gold microscopy (Figure 3). This analysis confirmed AQP1 localization in the apical and basal plasma membranes of mouse capillary endothelia (Figure 3). Quantitative immuno-gold electron microscopy (Figure 4) confirmed the results of the semi-quantitative immunoblotting showed in this study. There were significant differences in the numbers of gold particles/µm^2^ of AQP1 between 72 h fasted AQP7−/− mice and AQP7−/− and AQP7+/+ controls, with *p* < 0.01 (*n* = 3) and *p* < 0.05 (*n* =3), respectively. However, there were no statistically significant changes in anti-AQP1 immunogold labeling between fasted and control AQP7+/+ groups (n.s., *n* = 3; Figure 4).

Furthermore, AQP7 knock-out mice did not show a significant difference in serum glycerol level in control (AQP7+/+ = 431 ± 51 µmol/L and AQP7−/− = 459 ± 29) and in fasted (AQP7+/+ = 407 ± 41 µmol/L and AQP7−/− = 441 ± 34) conditions but demonstrated a serious loss of glycerol in the urine of AQP7−/− mice fasted for 72 h compared to the control (basal; 131 ± 13 µmol/L vs. fasted; 4002 ± 319 * µmol/L AQP7−/− mice; data presented in brackets are means ± SE, * *p* < 0.05).

## 3. Discussion

In this study, we investigated whether prolonged starvation causes regulation of AQP1 expression in capillaries of WAT of AQP7 KO mice. We found AQP1 to be expressed in WAT vessels of all studied genotypes (control mice: AQP7+/+, AQP7−/−; and fasted mice: AQP7+/+, AQP7−/−). Moreover, the abundance of AQP1 in the vessels was up-regulated in response to starvation. AQP7 KO mice (used in the present study) did not show a difference in the serum glycerol level in basic (control) or fasted conditions, but demonstrated a serious loss of glycerol in the urine, which is consistent with our previous observation [4] and with a study described by Sohara et al. [16]. The explanation of this phenomenon is that glyceroluria in AQP7 knock-out mice does not depend on a shift in plasma glycerol levels. This is, rather, the consequence of a change of glycerol reabsorption in the kidneys. Prolonged starvation had no effect on the adipocyte size in control or AQP7 knock-out mice, which is corroborated with our previous study [4], in which we described a co-expression of AQP1 with AQP7 in the capillaries of mouse WAT and that AQP7 expression in the capillaries rises in response to prolonged fasting [4]. The expression of AQP7 protein in the capillary endothelia of mouse WAT suggests a non-cell-autonomous role for AQP7 in promoting antihypertrophic effects [4].

Fasting causes triglycerides to be hydrolyzed into free fatty acids and glycerol, and then releases them from adipocytes into the blood stream by various transcellular mechanisms [4]. A pioneering study performed by Kishida et al. [12] has identified AQP7 as a potential aquaporin isoform associated with the adipose tissue. Recent studies with the use of two independent lines of AQP7 knock-out mice confirmed that AQP7 is a major channel needed for the release of glycerol from WAT [14,17]. Our research [4] using AQP7 KO mice confirmed the above-mentioned results, showing that AQP7 is a regulator of glycerol transport in mice. Moreover, it has been reported [2,3,18,19] that several other aquaporins, including AQP3, AQP5, AQP9, AQP10 and AQP11, are expressed and involved in glycerol transport in WAT of many species, including humans. Recently, AQP10 was found by Laforenza et al. [19] to be an alternative pathway for glycerol outflow from human and mouse WAT. The authors described the presence at least two co-working AQPs in WAT, AQP7 in both capillary plasma and adipocyte membranes of human and mouse WAT and only AQP10 in the adipocytes. Moreover, they found that *AQP10* silencing in humans differentiated adipocytes resulting in an approximately 50% decrease in osmotic water and glycerol permeability. Very recently, an interesting study was performed by Madeira et al. [20] on human AQP11 (an unorthodox AQP). The authors reported the expression of AQP11 in human adipocytes. Furthermore, using the 3T3-L1 cell model in which AQP11 was over-expressed in adipocytes, they found this protein acting both as a glycerol and water channel. In turn, Rojek et al. [21] found that AQP11 KO mice die early due to failure of the kidney. However, the generation of liver-specific AQP11 knock-out mice [21] has shown that they have normal plasma parameters and exposed only a slight fault in lipid handling. Starvation and re-feeding of the mice induced changes of the rough endoplasmic reticulum (RER) in the hepatocytes. Similar effects, especially a fall in glucose levels, were observed after the administration of pure protein and various amino acids [21]. The authors concluded that *AQP11* in the mouse liver is an important factor in RER homeostasis.

The expression of AQP1 is well-documented in the microvascular endothelial cells and its expression may be regulated by several factors. For example, Lai et al. [22] found that osmotic agents such as mannitol and glucose up-regulated AQP1 and AQP3 mRNA and protein expression [23] in human peritoneal mesothelial cells. In another experiment performed with the use of AQP1 KO mice, Ma at el. [24] found that the mice processed dietary fat abnormally when fed a high-fat diet. Our current study demonstrates that AQP1 protein expression is up-regulated by 2.5- to 3-fold in the capillaries of WAT in the 72 h starved AQP7 knock-out mice.

Aquaporin 5 has recently been revealed by Madeira et al. [3] as a functional water channel in human adipocytes and the presence of this protein may be associated in water homeostasis and differentiation within adipocytes. In both the present and previous experiments [4], prolonged 72 h starvation did not have an effect on the adipocyte size in the control or AQP7 knock-out mice. Recent research performed by Wu et al. [25] described AQP5 as a factor participating in controlling AQP2 in the kidney of mice with a deficiency of Dot1l histone H3K79 methyltransferase (Dot1l) in renal AQP2-expressing cells and in patients with diabetic nephropathy. The study suggests that up-regulated AQP5, perhaps by reducing AQP2 membrane localization, may contribute to polyuria. Moreover, Amlal et al. [26] found that fasting impairs the urinary concentration ability in rat results, specifically from the down-regulation of AQP2 in kidney. Our recent in vitro study [27] showed AQP1 expression in the vessels and AQP5 in the epithelial cells of the uterus of cyclic gilts and that their specific localization and regulation by steroid hormones, arachidonic acid-derivatives and cAMP may influence the transcellular water movement between blood vessels and uterine lumen.

It is well known that AQPs, in response to osmotic gradients, play a crucial role in the water balance of cells and tissues to maintain their function. AQP1 and AQP7 are co-expressed in the capillary membranes of the mouse white adipose tissue. A deficiency of AQP7 and fasting cause the up-regulation of AQP1 in the capillary endothelia of the mouse WAT to maintain the proper function of this tissue.

In conclusion, the current research delineated the up-regulation of AQP1 expression in the capillary endothelium of WAT after prolonged fasting in AQP7 KO mice and indirectly provided novum for its role in maintaining local water homeostasis within the WAT in mice. This finding suggests that an interaction of AQP isoforms is required for key cellular functions within the mouse WAT. Nevertheless, further studies are necessary to explain how AQP1 isoforms cooperate with other AQPs (e.g., AQP5) during different stages of metabolism in the mouse WAT.

## 4. Materials and Methods

### 4.1. Animal Models

The animal protocols were approved and the license for using experimental animals was issued by the Danish Ministry of Justice. The mice were kept with a 12:12 h artificial light-dark cycle, a temperature of 21 ± 2 °C and a humidity of 55% ± 2%, with free access to tap water and were maintained on a standard rodent diet (1324 pellets, Altromin, Lage, Germany). The generation of AQP7 KO mice has been described previously [4]. For the experiment with 72 h fasting, male mice with an average weight of 25 g were divided into four groups matched for body weight: AQP7−/− control (*n* = 5), AQP7+/+ control (*n* = 5), AQP7−/− 72 h fasting (*n* = 5), and AQP7+/+ 72 h fasting (*n* = 5). For the fasting group, food was removed 72 h before death. Tissue samples were taken for immunoblotting, immunohistochemistry and immunoelectron microscopical analysis. Adipose tissue was taken from the epididymis surroundings.

### 4.2. SDS-PAGE and Immunoblotting

The tissues were immediately placed in an ice-cold dissection buffer (0.3 M sucrose, 25 mM imidazol, 1 mM EDTA in ddH_2_O, pH 7.2, containing 8.4 μM leupeptin, 0.4 mM pefabloc). After dissection, the tissue samples were homogenized using an ultra Turrax T8 homogenizer (IKA Labortechnik, Staufen, Germany) and centrifuged at 4000× *g* for 15 min at 4 °C. The supernatant added was an SDS-containing sample buffer, giving a final concentration of 62 mM Tris (hydroxymethyl)-aminomethane, 0.1 M SDS, 8.7% glycerol, 0.09 mM bromophenol blue and 0.04 M dithiothreitol (DTT), pH 6.8. The protein samples were heated for 5 min at 90 °C and stored at −20 °C until use. The samples were heated to 37 °C and loaded into 12% polyacrylamide gels and the proteins were separated by electrophoresis. For semi-quantification, the total protein amount in each sample was adjusted by staining with Gelcode Coomassie Blue Stain Reagent (Bie and Berntsen A/S, Åbyhøj, Denmark) to ensure equal loading. The proteins of subsequent gels were electro-transferred onto nitrocellulose membranes (Hybond ECL RPN3032D, Amersham Pharmacia Biotech, Little Chalfont, UK) for 1 h at 100 V. The membranes were blocked with 5% milk in PBS-T (80 mM Na_2_HPO_4_, 20 mM NaH_2_PO_4_, 100 mM NaCl, pH 7.5 and 0.1% *v*/*v* Tween 20) for 1 h. After being washed, the membranes were incubated overnight at 5 °C with anti-AQP1 antibody. The antibody-to-mouse AQP1s (RA3391/2353) were previously characterized [16]. The membranes were washed and incubated with horseradish peroxidase-conjugated goat anti-rabbit IgG secondary antibody (Dako A/S, Glostrup, Denmark) in PBS-T for 1 h. After being washed with PBS-T, the bound antibody was detected by ECL chemiluminescence kit (Amersham Bioscience, Pittsburgh, PA, USA). The luminescence was detected on a light-sensitive Hyperfilm (Amersham Bioscience). Semi-quantification of the immunoreactive proteins was performed using a scanner (Duoscan f40, Agfa, Glostrup, Denmark) and Scion Image software (Available online: www.scioncorp.com) after background subtraction. The band intensities were measured within the linear range.

### 4.3. Immunohistochemistry for Light Microscopic Examination

Tissues were fixed by retrograde perfusion via the aorta with 3% paraformaldehyde, in 0.1 M cacodylate buffer, pH 7.4, as reported previously [4]. Briefly, paraffin-embedded tissue sections (2-μm thickness) were dehydrated and finally embedded in paraffin. The staining was carried out using indirect immunoperoxidase. The sections were de-waxed and rehydrated. For immunoperoxidase labeling, endogenous peroxidase was blocked by 0.5% H_2_O_2_ in absolute methanol for 10 min at room temperature. To reveal antigens, sections were submerged in 1 mM Tris solution (pH 9.0) supplemented with 0.5 mM EGTA and heated in a microwave oven at 650 W for 6 min and then at 350 W for 10 min as described previously. Non-specific binding of IgG was prevented by incubating the sections in 50 mM NH_4_Cl for 30 min, followed by blocking in PBS supplemented with 1% BSA, 0.05% saponin, and 0.2% gelatin. Sections were incubated overnight at 4 °C with primary antibody diluted in PBS supplemented with 0.1% BSA and 0.3% Triton X-100. Antibody-to-mouse AQP1 (RA3391/2353) were previously characterized [28]. The sections were rinsed with PBS supplemented with 0.1% BSA, 0.05% saponin, and 0.2% gelatin and then incubated with horseradish peroxidase-conjugated secondary antibody (P448, 1:200, Dako, Glostrup, Denmark). Labeling was visualized by 0.05% 3,3-diaminobenzidine tetrahydrochloride (DAB). The microscopy was carried out using a Leica DMRE light microscope (Heidelberg, Germany).

### 4.4. Immunoelectron Microscopy of Mouse Capillaries

The tissues were prepared by freeze substitution and subjected to immunoelectron microscopical analysis as described previously [4]. Briefly, ultrathin Lowicryl HM20 sections were incubated overnight at 4 °C with anti-AQP1 antibodies and visualized with goat anti-rabbit IgG conjugated to 10 nm colloidal gold particles at 1:50 (GAR.EM10; BioCell Research Laboratories, Cardiff, UK). Sections stained with uranyl acetate and lead citrate were examined with a Philips CM100 or Philips Morgagni electron microscope (Philips, Eindhoven, The Netherlands). Quantitation of the immuno-gold labeling was performed on electron micrographs from capillaries from three different animals within each group. The number of gold particles associated with the membranes within single cells was determined. Quantitation of the electron micrograph results was carried out using the iTEM, FIVE Digital Imaging Solutions, based on the analySIS Platform (Olympus, Tokyo, Japan).

### 4.5. Plasma/Urine Glycerol Measurements

Mice were anesthetized and blood samples were collected from the right ventricle of the heart and plasma was analyzed for glycerol and free fatty acids (FFA). The FFA was measured by NEFA C KIT from Wako Chemicals GmbH (Richmond, VA, USA), detected by Elisa reader at 540 nm. Urine samples were obtained by placing the mice in metabolic cages.

### 4.6. Statistics

Data are expressed as means ± SE. Statistical analysis between groups was made by one-way ANOVA with Tukey-Kramer multiple comparisons *p* values <0.05 were considered statistical significant.

## Abbreviations

AQPs	Aquaporins
AQP7 KO	AQP7 knock-out mice
cAMP	Cyclic adenosine monophosphate
Dot1l	Dot1l histone H3K79 methyltransferase
n.s.	non significant
RER	Rough endoplasmic reticulum
WAT	White adipose tissue
